# Information-theoretic gene-gene and gene-environment interaction analysis of quantitative traits

**DOI:** 10.1186/1471-2164-10-509

**Published:** 2009-11-04

**Authors:** Pritam Chanda, Lara Sucheston, Song Liu, Aidong Zhang, Murali Ramanathan

**Affiliations:** 1 Department of Computer Science and Engineering, State University of New York, Buffalo, NY, USA; 2 Department of Biostatistics, State University of New York, Buffalo, NY; 3 Division of Cancer Prevention and Control, Roswell Park Cancer Institute, Buffalo, NY, USA; 4 Department of Pharmaceutical Sciences, State University of New York, Buffalo, NY, USA

## Abstract

**Background:**

The purpose of this research was to develop a novel information theoretic method and an efficient algorithm for analyzing the gene-gene (GGI) and gene-environmental interactions (GEI) associated with quantitative traits (QT). The method is built on two information-theoretic metrics, the *k*-way interaction information (KWII) and phenotype-associated information (PAI). The PAI is a novel information theoretic metric that is obtained from the total information correlation (TCI) information theoretic metric by removing the contributions for inter-variable dependencies (resulting from factors such as linkage disequilibrium and common sources of environmental pollutants).

**Results:**

The KWII and the PAI were critically evaluated and incorporated within an algorithm called CHORUS for analyzing QT. The combinations with the highest values of KWII and PAI identified each known GEI associated with the QT in the simulated data sets. The CHORUS algorithm was tested using the simulated GAW15 data set and two real GGI data sets from QTL mapping studies of high-density lipoprotein levels/atherosclerotic lesion size and ultra-violet light-induced immunosuppression. The KWII and PAI were found to have excellent sensitivity for identifying the key GEI simulated to affect the two quantitative trait variables in the GAW15 data set. In addition, both metrics showed strong concordance with the results of the two different QTL mapping data sets.

**Conclusion:**

The KWII and PAI are promising metrics for analyzing the GEI of QT.

## Background

The clinical presentation of many common complex diseases causing morbidity and mortality are associated with deviations from the population distributions of important quantitative traits (QT). For example, in hypertension and non-insulin dependent diabetes, the disease processes increase the QT, blood pressure and blood glucose, respectively. For many diseases, threshold values of QT are the basis for the diagnostic criteria for the diseases. However, obtaining an in-depth understanding of genetic and environmental determinants of QT such as weight, height and lifespan in healthy populations can also be important scientific questions. The regulation of many QT is typically complex and involves interactions among many genes as well as endogenous and exogenous factors [1,2]. For example, genes in pathways regulating appetite, metabolism, hormones and adipokines may interact with environmental factors such as diet and exercise to determine body weight. Nonetheless, the successful identification of the critical gene-environment interactions (GEI) involved in QT such as body weight can provide the scientific basis for preventative public health measures to reduce the exposure of individuals to the modifiable environmental variable/s associated with increased risk.

Information theoretic methods have considerable promise for enhancing single nucleotide (SNP), gene-gene interaction (GGI) and GEI analysis [3-6]. The Kullback-Leibler divergence (KLD), an information theoretic measure of the 'distance' between two distributions, has been proposed for 2-group comparisons such as those used to evaluate ancestry informative markers [7-9], as a multi-locus linkage disequilibrium (LD) measure to enable identification of TagSNPs [6] and for analytical visualization [4,5]. Entropy-based statistics to test for allelic association with a phenotype [10-12] and for two-locus interactions have also been proposed [13]. Information theoretic extensions of the KLD allow measurement of complex multivariate dependencies among genetic variations and environmental factors without complex modeling and could enable powerful and intuitive methodology for GGI and GEI analyses to be developed [14,15].

While there is now considerable evidence demonstrating the usefulness of information theoretic methods for identifying the interactions associated with discrete and binary phenotypes, to our knowledge, information theoretic approaches have not been reported for analyzing the GGI and GEI associated with QT. This report proposes an information-theoretic approach for identifying associations of GEI and GGI with a QT.

## Methods

### Terminology and Representation

#### Definition of Interaction

In our information theoretic framework, we use the *K*-way interaction information (KWII) [16,17], which is defined and described in detail below, as the measure of interaction information. We operationally define "*for each variable combination containing the QT phenotype, a positive KWII value indicates the presence of an interaction, negative values of KWII indicates the presence of redundancy and a KWII value of zero denotes the absence of K-way interactions*".

The methods in this paper are applicable to both GEI and GGI analyses and henceforth, we will simply use the term GEI to refer to both. The underlying terminology and representation for this paper was developed in our earlier publications [14,15] but is concisely recapitulated here.

The operational definition can yield results that are difficult to interpret in the presence of variables that are completely redundant with each other because an even number of completely redundant variables will result in a positive KWII. We address these issues in detail in Discussion.

#### Entropy

The entropy, *H*(*X*), of a discrete random variable *X *can be computed from its probability mass function, *p*(*x*), using the Shannon entropy formula:

The entropy, *H*(*X*), of a continuous random variable *X *can be computed from its probability density function, *f*(*x*), using the formula:

#### K-way interaction information

For the 3-variable case involving two genetic or environmental variables denoted by *A *and *B*, and the QT phenotype denoted by *P*, the KWII is defined in terms of entropies of the individual variables, *H(A), H(B) *and *H(P) *and the entropies, *H(AB), H(AP), H(BP) *and *H(ABP)*, of the combinations of the variables:

For the *K*-variable case on the set *v *= {*X*_1_, *X*_2_, ..., *X*_*K*_, *P*}, the KWII can be written succinctly as an alternating sum over all possible subsets *T *of *v *using the difference operator notation of Han [18]:

The number of genetic and environmental variables *K *in a combination is called the order of the combination. The KWII quantifies interactions by representing the information that cannot be obtained without observing all *K *variables and the QT phenotype *P *at the same time [16,17,19,20]. The KWII of a given combination of variables is a parsimonious interaction metric in that it does not contain contributions arising from the KWII of other lower order combinations (i.e., the subsets of the *K*-way variable combination).

#### Total Correlation Information (TCI)

For the 3-variable case, the TCI [21] is defined in terms of entropies of the individual variables *H(A), H(B) *and *H(P) *and the entropy of the joint distribution *H(ABP)*:

For the case containing *K *genetic or environmental variables and the QT on the set v = {*X*_1_, *X*_2_, ..., *X*_*K*_, *P*}, the TCI, can be expressed as the difference between the entropies of the individual variables *H(X*_*i*_) and the entropy of the joint distribution *H(X*_1_*X*_2_... *X*_*K*_*P)*.

Similarly for *K *genetic or environmental variables combinations not including the QT:

The TCI is the amount of information shared among the variables in the set; equivalently, it can be viewed a general measure of dependency. A TCI value that is zero indicates that knowing the value of one variable tells you nothing about the others, i.e., that the variables are independent. The maximal value of TCI occurs when one variable is completely redundant with the others.

### Phenotype-Associated Information

The *phenotype-associated information *(PAI) is obtained from the TCI for the overall dependency among the genetic and environmental variables and the phenotype variable by removing the TCI contributions representing the interdependencies among the genetic and environmental variables only. The interdependencies among these variables can be caused for example, by LD or by correlated source patterns of pollutant exposures. Accordingly, PAI is defined by:

In the above equation, the genetic and environmental variables are denoted by the *X*_1_, *X*_2_,..., *X*_*K*_, and the quantitative trait is denoted by *P*.

In the PAI definition, the *TCI(X*_1_, *X*_2_,..., *X*_*K*_, *P) *term represents the overall dependency among the genetic and environmental variables and the phenotype whereas the *TCI(X*_1_, *X*_2_,..., *X*_*K*_) term represents the interdependencies among the genetic and environmental variables in the absence of the phenotype variable.

Our approach utilizes the KWII as the principal measure of the GEI. However, we employ the PAI to facilitate efficient searching of the combinatorial space. Although the KWII is a parsimonious measure of interaction for the variable combination of interest alone because it does not contain contributions from lower-order interactions, KWII computations on the entire combinatorial space are computationally intractable because they require the entropies of all subsets. In addition, the KWII cannot be used for hill climbing algorithms because it takes on both positive and negative values depending on the nature of the interaction but the values cannot be used to direct the search process.

In contrast, the PAI is appropriate for hill climbing algorithms as it is always greater than or equal to zero and increases monotonically with increased combination size. PAI calculations require only individual and joint entropies and are computationally far more tractable than KWII calculations. In addition to these desirable properties, the PAI contains useful information regarding the KWII because it represents the cumulative phenotype-associated synergy present in all subset combinations of the variables *X*_1_, *X*_2_, ..., *X*_*K *_and *P*. In Results, we will demonstrate that both the KWII and PAI metrics are effective and efficient for GEI analysis.

### Extension to Quantitative Traits

The expressions for KWII and PAI are general and can be used for QT and categorical phenotypes. However, the specific forms are necessary for the entropy of the QT and the joint entropy of the QT with discrete variables resulting from the genetic variants, environmental variables and their combinations.

The entropy *H*(*Z*) of a normally distributed variable, *Z*, with mean *μ *and standard deviation *σ *is [22]:

Note that the entropy, *H*(*Z*), of a normally distributed variable is independent of the mean *μ*.

For GGI and GEI analysis in this report, we are interested in *H*(*X*, *P*), the entropy of the joint distribution of the QT, *P*, and a discrete variable *X*, representing, e.g., genetic variants or environment variables of interest or their combinations.

Therefore:

We assume that the QT, *P*, given *X *= *x*, is normally distributed *N*(*μ*_*x*_, *σ*_*x*_). By expanding, simplifying and substitution, we obtain:

The *H(X) *term contains only discrete variables and as a result, this entropy can be computed using the Shannon entropy formula:

These equations for the entropy of the QT, the entropy of discrete variable combinations and the joint entropy of the QT and discrete variable enable computation of the KWII and the PAI for GEI analysis of QT.

### The CHORUS Algorithm

**Figure 1:**
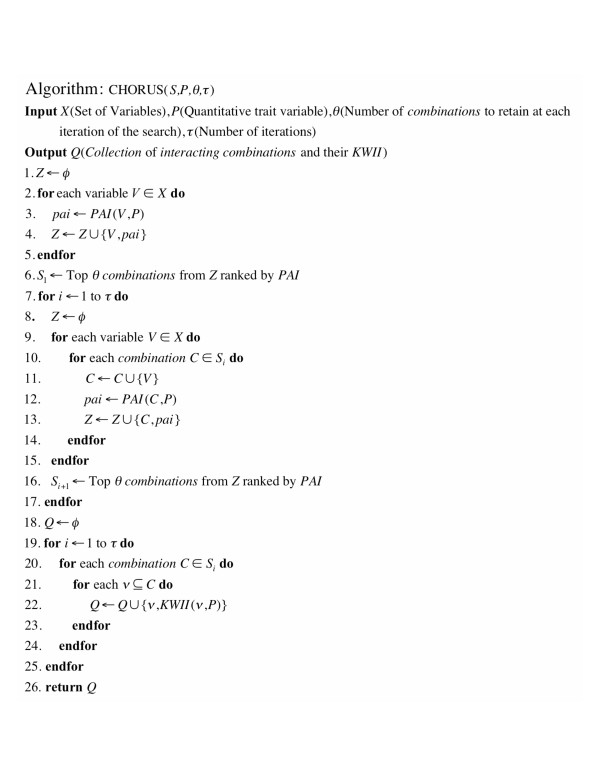
**Pseudocode for the CHORUS algorithm for detecting GEI associated with quantitative traits**.

The CHORUS Algorithm is a computationally efficient approach for GEI analysis of QT that is based on our earlier work with discrete phenotypes [15]. The pseudocode for CHORUS is shown in Figure 1.

The CHORUS Algorithm employs the PAI to iteratively search for combinations of genetic variations and environmental variables related to the QT. The increases in the PAI are used to guide the search process in a hill-climbing algorithm that identifies promising subsets of combinations potentially involved in GEI with the QT. The KWII values necessary for assessing GEI in our method are then computed for the promising subsets of combinations identified.

Let *X *= {*X*_1_, *X*_2_, ..., *X*_*n*_} be the set of all genetic/environmental variables and *P *be the QT of interest. The algorithm takes as input *X*, *P*, and algorithm parameters *θ *and *τ*, which represent the number of combinations retained in an iteration of the search and the number of iterations, respectively.

We start by calculating *PAI*(*X*_*i*_, *P*) ∀*i *∈ 1 ... *n*. We retain the *θ *combinations with the highest values of *PAI *for use in the next iteration. Let this set of variables be denoted by *S*_1_. In the next step, we calculate *PAI*(*X*_*i*_, *X*_*j*_, *P*) ∀ *X*_*i*_∈ 1... *n*, (*j *≠ *i*) and again retain the *θ *combinations with the highest values of *PAI*(*X*_*i*_, *X*_*j*_, *P*) in set *S*_2_. The above steps are repeated *τ *times. Thus, we greedily search for combinations containing up to *τ *variables that have higher values of PAI. This search process identifies regions in the combinatorial space with the strongest KWII-based interactions among the variable combinations examined. Finally, for each combination *C *identified by the above search steps, we calculate the *KWII*(*v*, *P*) ∀ *v *⊆ *C *to identify the interacting combinations, {*v, P*}.

The simulations and CHORUS implementations were built in-house and written in the Java 1.5 programming language. The COLT high-performance computing library from CERN (acs.lbl.gov/~hoschek/colt) was used for numerical functions including random variate generators.

### Simulated Data

Simulated data sets were used to examine: 1) the relationship between LD, KWII and PAI in the presence of association with a quantitative trait, 2) the correlation between effect size and KWII values and, 3) the power of KWII to detect association across various effect sizes.

#### Approaches Common for All Simulated Data Sets

All simulated data sets contained six diallelic SNPs with the three possible genotypes. The genotypes at each locus were assumed to be in Hardy-Weinberg equilibrium proportions. The Simulated Data Sets 1, 2 and 3 consisted of 2 interacting SNPs, SNP(1) and SNP(2), that were associated with the QT. An additional 4 null SNPs, SNP(3) through SNP(6), were simulated. The alleles of SNP 1 were labeled *A*_1 _and *A*_2_, and those of SNP 2 were labeled *B*_1 _and *B*_2_. All SNPs had a minor allele frequency (MAF) of 0.5.

**Figure 2:**
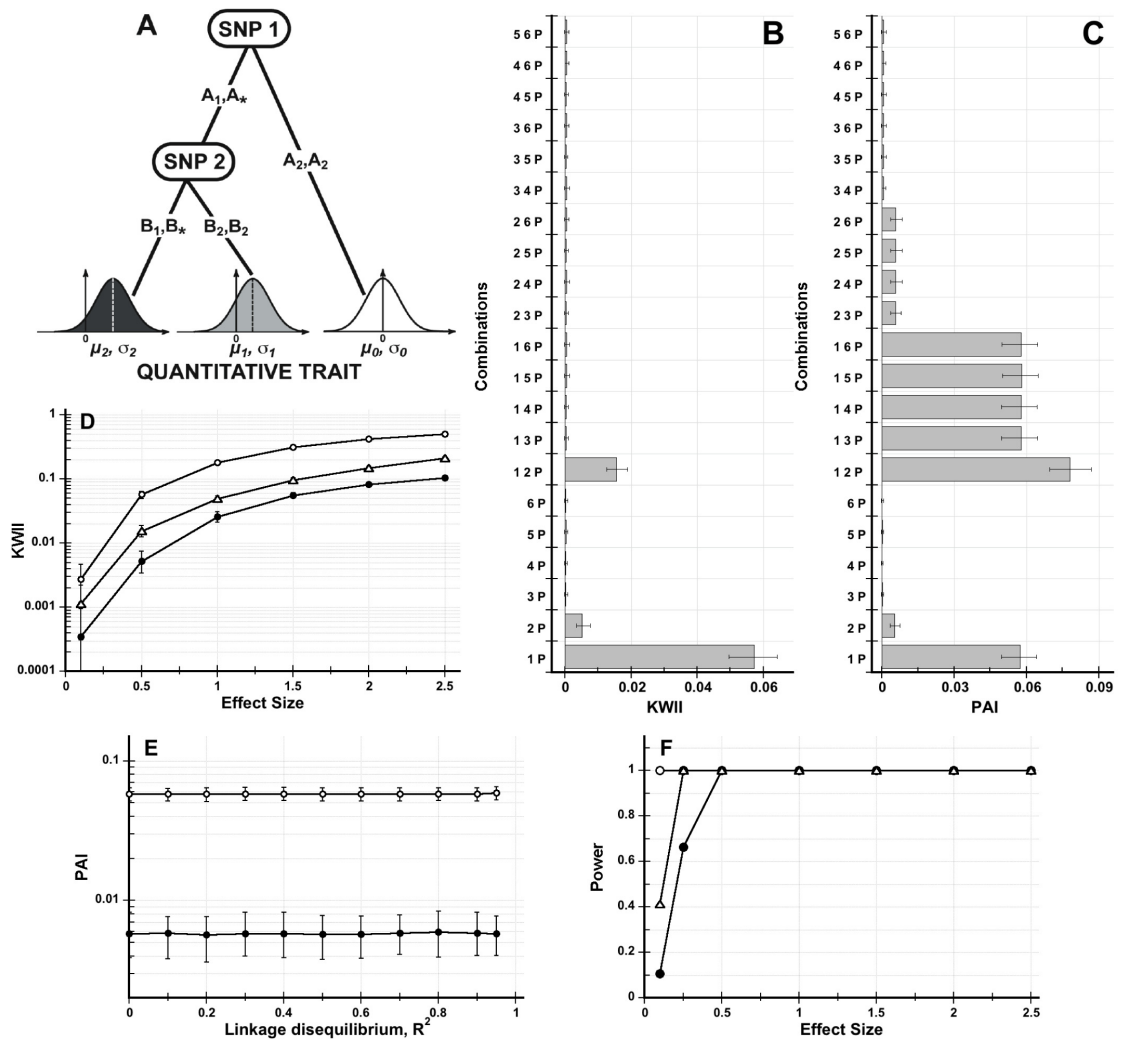
**Figure 2A shows the interaction model for Simulated Data Set 1**. The SNP variables, *SNP 1 *and *SNP 2 *sequentially interact (the comma in 2, 3 indicates the Boolean *OR *operation) to determine the QT level. Figure 2B and 2C are the corresponding KWII and PAI spectra for an effect size of 0.5. Figure 2D shows the dependence of KWII on effect size for the {*1*, *P*} (open circles), {*2*, *P*} (filled circles) and {*1*, *2*, *P*} (open triangles). Figure 2E shows the PAI for the {*1, 3, P*} (open circles) and {*2, 4, P*} (filled circles) combinations when pairwise LD between SNPs 1 and 3 and SNPs 2 and 4 were varied. The error bars in Figures 2B-E represent the 95^th ^percentile and 5^th ^percentile values. Figure 2F shows the power of the KWII as a function of effect size for the {*1*, *P*} (open circles), {*2*, *P*} (filled circles) and {*1*, *2*, *P*} (open triangles) combinations.

**Figure 3:**
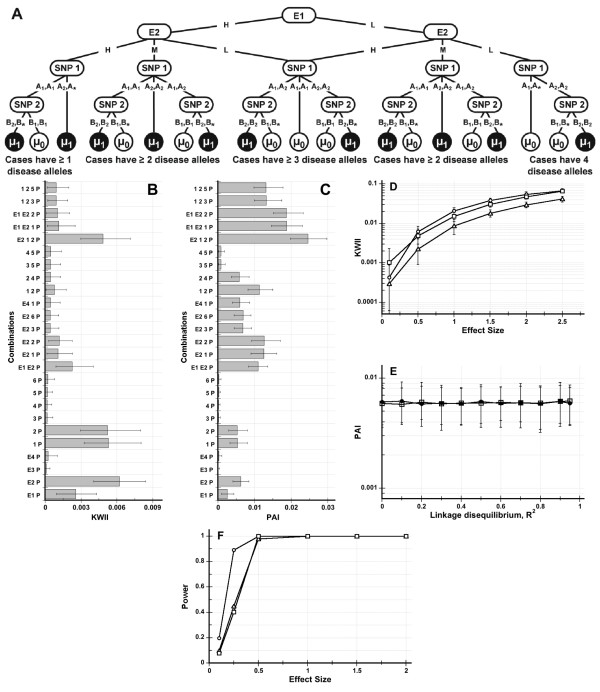
**Figure 3A shows the interaction model for Simulated Data Set 2**. The asterisk in a genotype represents a "wild card" indicating that either allele is allowable. Figure 3B and 3C are the KWII and PAI spectra for an effect size of 0.5. All the one-variable containing combinations and the 10 two-variable and 5 three-variable combinations with the highest KWII values are shown. Figure 3D shows the dependence of KWII on effect size for the {*E2*, *P*} (open circles), {*E1*, *E2*, *P*} (open triangles) and {*E2, 1*, *2*, *P*} (open squares). Figure 3E shows the PAI for the {*1, 3, P*} (open squares) and {*2, 4, P*} (filled circles) combinations when pairwise LD between SNPs 1 and 3 and SNPs 2 and 4 were varied. The error bars in Figures 3B-E represent the 95^th ^percentile and 5^th ^percentile values. The symbols and the error bars in Figure 3E are difficult to distinguish because the values for both combinations were nearly equal. Figure 3F shows the power of the KWII as a function of effect size for the {*E2*, *P*} (open circles), {*E1*, *E2*, *P*} (open triangles) and {*E2, 1*, *2*, *P*} (open squares) combinations.

The GEI models for Simulated Data Sets 1 and 2 are shown in Figures 2A and 3A, respectively. Based on genotype or genotype-environmental combination, the QT value for each subject was a normally distributed random variate with a mean of either *μ*_0 _= 0 or *μ*_1 _= 0.5. The corresponding standard deviation values of the QT distributions, *σ *= *σ*_0 _= *σ*_1 _were set equal. The effect size Z was defined as the ratio (*μ*_1 _- *μ*_0_)/*σ*.

Samples composed of 5000 individuals with randomly varying genotypes and environmental exposures consistent with the underlying GEI models were simulated. The computations for all three Simulated Data Sets were performed with CHORUS input parameter values of *τ *= 3 and *θ *was set to include all possible combinations at each iteration.

To obtain *p*-values of the KWII, the null distribution of the KWII of each combination was obtained from 5000 random permutations of the QT values for each simulated data set. The permutations for each combination were conducted independently. The raw *p*-value of the KWII of a combination was ascertained as the fraction of the permuted KWII values that were equal to or greater than the observed KWII value. The *p*-values were not corrected for multiple testing.

The 5^th ^and 95^th ^percentiles of the KWII and PAI, representing the variations due to sampling, were obtained from 100 independent repetitions of the simulation procedure. In forming confidence intervals we did not consider multiple testing. The number of potential tests for all one-way, two-way and three-way combinations was 41 each for Simulated Data Sets 1 and 3 and 175 for Simulated Data Set 2.

For the simulations summarized in Figures 2B-2C, 3B-3C, the effect size was set to 0.5. For the simulations in Figure 2D and 3D, the effect size was varied from 0.5 to 2.5 in increments of 0.5 by varying the standard deviation of QT distribution. Linkage disequilibrium (LD) was assumed to be absent in the simulations in Figures 2B-D and 3B-D. In Figure 2E and 3E, we incorporated LD between the SNP 1-SNP 3 and the SNP 2-SNP 4 pairs; the *R*^2 ^values for both pairs was set to be equal and eleven *R*^2 ^values ranging from 0 to 0.95 were studied.

#### Simulated Data Set 1, Two-SNP Interactions

The mean QT values for each genotype were based on the GGI model in Figure 2A. The standard deviation *σ*_2 _was set to *σ*, and *μ*_2 _= 1.

#### Simulated Data Set 2, Multi-SNP and SNP-Environment Interactions

The mean QT values for each genotype were based on the GEI model in Figure 3A. There were four environmental variables, *E(1) *through *E(4)*, in addition to the six SNPs. The interacting environmental variables, *E(1) *and *E(2)*, were associated with the QT; *E(3) *and *E(4) *were null environmental variables. The percentage of subjects in low (*L*) and high (*H*) exposure categories of *E(1) *and *E(3) *were each 50%; the percentage of subjects in low (*L*), medium (*M*) and high exposure (*H*) categories of *E(2) *and *E(4) *were 25%, 50% and 25%, respectively.

#### Simulated Data Set 3, Two Locus Model with Pure Epistasis Interactions

The Simulated Data Set was selected to contain pure epistasis interactions without main effects present, i.e., it does not contain QT variation that is attributable to any individual locus and requires the combined presence of two loci for explaining the QT variation.

In contrast to Simulated Data Sets 1 and 2, which were based on biological models of causation, the simulations for Simulated Data Sets 3 were based on models of pure epistasis that were evaluated by Culverhouse [23]. The QT values for each genotype were random variates drawn from *N*(*μ*_1_, *σ*_1_) with frequency corresponding to the probability in the following table and from *N*(*μ*_0_, *σ*_0_) otherwise:

#### Power Calculations

**Figure 4:**
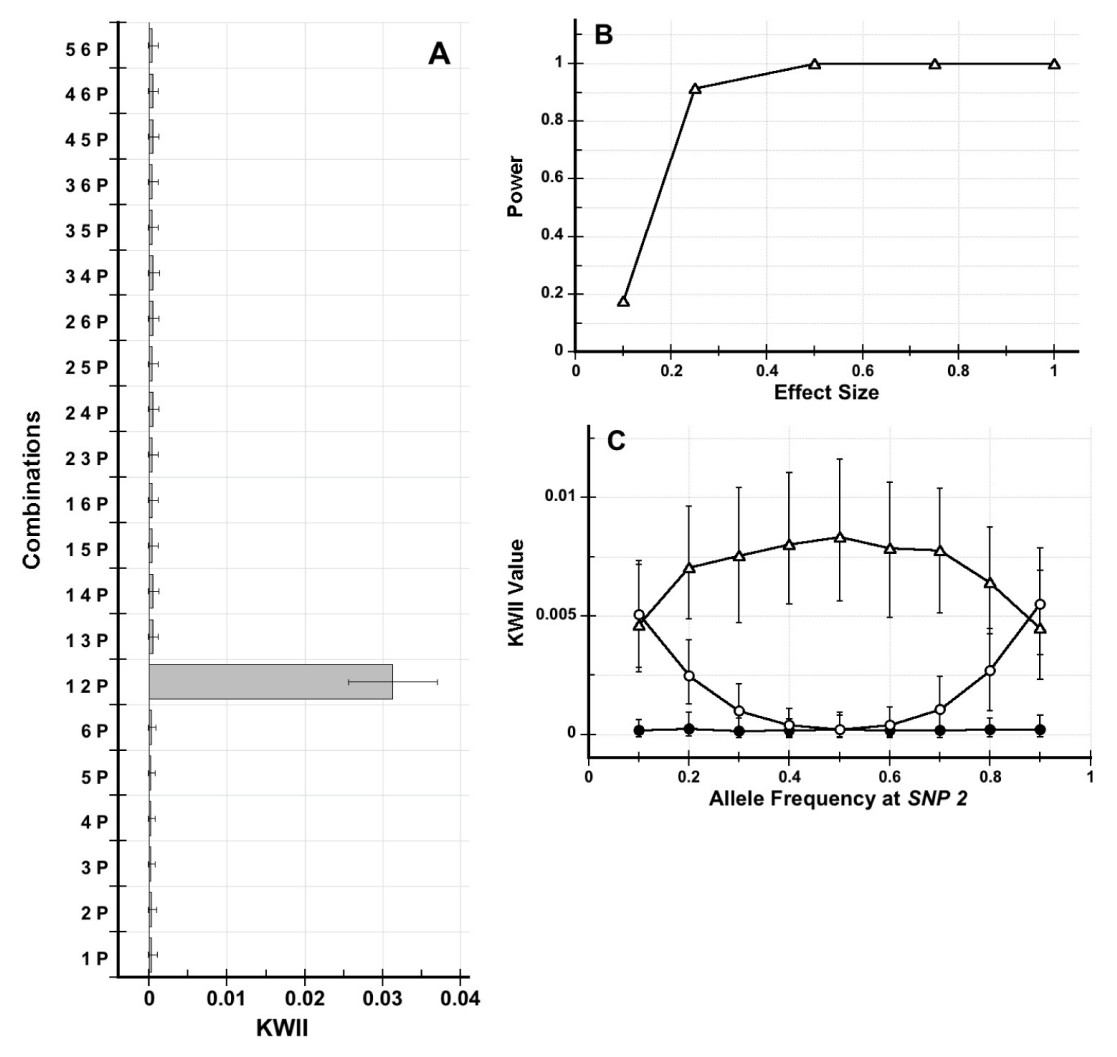
**Figure 4A shows the KWII spectrum for the two-locus models with pure epistasis interactions, respectively**. Figure 4B shows the power of the KWII value of the {*1*, *2*, *P*} combination in the two-locus model for different effect sizes. Figure 4C shows the dependence of the KWII values in the two-locus model for the {*1*, *P*} (open circles), {*2*, *P*} (filled circles) and {*1*, *2*, *P*} (open triangles) combinations on the allele frequency at SNP 2.

For the power calculations in Figures 2F, 3F and 4B, 1000 independent simulations were conducted for each of the following effect sizes: 0.1, 0.25, 0.5, 1, 1.5, 2, and 2.5 by changing the standard deviation. The sample sizes were assumed to be 5000. The distribution of the KWII for an effect size of zero was obtained and its 95^th ^percentile value was computed. Positive values of KWII indicate the presence of interactions and accordingly, power at the non-zero effect size values was defined as the fraction of the simulations whose KWII value exceeded the 95^th ^percentile value of the KWII distribution for the zero effect size.

### Analysis of Public Domain Data Sets

The computations for all three public domain data sets were performed with CHORUS input parameters values of *θ *= 50 and *τ *= 2.

We did not correct for multiple testing; instead, we present the number of tests for all one and two-way combinations for each dataset and the top 25 unadjusted *p*-values and corresponding KWII values for one and two-way combinations. Given only one dataset is based on simulated data and the other two are real datasets in which the authors adjusted for multiple testing using different methods we felt adjusting further confounded the comparisons and thus simply present the rank unadjusted values for scrutiny.

#### GEI Analysis of Genetic Analysis Workshop 15 Data

The data for Problem 3 of Genetic Analysis Workshop 15 (GAW15) were obtained from the GAW site http://www.gaworkshop.org/gaw15data.htm and used with permission. These data consist of 100 replicates of simulated data that are modeled after the rheumatoid arthritis (RA) data [24]. The interactions between the diallelic SNP loci and variables modeled in the data set are summarized in Table 1. The biomarkers, anti-cyclic citrullinated peptide antibody (Anti-CCP), immunoglobulin M (IgM) are defined for the cases only.

**Table 1:** Effects of major trait loci and covariates in the GAW15 data set.

Locus	Chr	SNP #	Phenotype	Effects
DR	6	152-155	RA	Affects risk of RA
A	16	30-31	RA	Controls effect of DR on RA risk
B	8	442	RA	Controls effect of smoking on RA risk
C	6	152-155	RA	Increases RA risk only in women
D	6	161-162	RA	Rare allele increases RA risk 5-fold
**E**	**18**	**268-269**	**RA, Anti-CCP**	**Affects DR on anti-CCP and increases RA risk**
**F**	**11**	**387-389**	**IgM**	**QTL for IgM**
G	9	185-186	Severity	25% QTL for severity
H	9	192-193	Severity	25% QTL for severity
Age	-		RA	Affects RA risk through smoking and sex ratio
Sex	-		RA	Affects RA risk with Locus C
**Smoking**	-		**RA, IgM**	**Affects RA risk with Locus B and IgM**.

The loci associated with IgM and Anti-CCP QT are shown in bold.

For our analysis, which aimed to evaluate the effectiveness of CHORUS, we have used the set of 9187 SNPs along with sex, age and smoking status as covariates. We refer to this data set as the "10K GAW15 Dataset." We conducted separate analyses with Anti-CCP and IgM as QT of interest. The Anti-CCP and IgM variables were logarithm (base 10) transformed prior to analysis. The Age variable was binned into five intervals of equal width. Although haplotype-phase information was provided, we chose to not include it and treated the data as unphased genotype data.

#### Analysis of High Density Lipoprotein and Atherosclerosis Data

Female B6 mice have low levels of plasma high-density lipoprotein (HDL) and are susceptible to atherosclerosis whereas female 129 mice have high plasma HDL levels and are relatively resistant. This data set contains genotypes, HDL concentrations and size of aortic fatty streak measurements for 294 female F2 intercross progeny (derived from the C57BL/6 (B6) and 129 strains of inbred mice) that were fed a high-fat diet for 14 weeks [25]. The mice were genotyped with 88 simple sequence length polymorphic (SSLP) markers and subsequently 23 additional SSLP markers in the QTL regions were added. The data were obtained from the Center for Genome Dynamics at the Jackson Laboratory http://cgd.jax.org/nav/qtlarchive1.htm.

We analyzed the HDL and atherosclerotic aortic fatty streak lesion size as QT of interest. The atherosclerotic aortic fatty streak lesion size variable was logarithmically transformed (base 10) prior to analysis.

#### Comparative Genomics Analysis

We also conducted mouse-human comparative genomics analysis to evaluate the biological significance of the markers identified by our method. The list of human HDL and atherosclerosis QTL were obtained from [26] and [25,27], respectively. The coordinates of the QTL were converted to latest release (hg18, NCBI Build 36.1) of the human genome assembly based on the UCSC genome browser [28]. The mouse marker regions with peak position ± 16 Mbp (the average HDL [26] and Atherosclerosis [29] QTL is 32 Mbp) were retrieved from the latest mouse genome assembly (mm9, NCBI Build 37). The human homologous regions of mouse markers were obtained by parsing the chain alignment of mouse (mm9)-to-human (hg18) genome [30,31].

#### Analysis of Ultra-violet (UV) Light-Induced Immunosuppression Data

This data set contains results from a genome-wide scan using MIT microsatellite markers and was obtained from F1 backcross mice derived from the low susceptibility BALB/c and high susceptibility C57BL/6 strains of mice that were tested for systemic UV light-induced immunosuppression of a contact hypersensitivity response [32]. The data were downloaded from the Center for Genome Dynamics at the Jackson Laboratory http://cgd.jax.org/nav/qtlarchive1.htm. The data contain 64 markers and sex was included as a factor. The percent immunosuppression of the contact hypersensitivity responses in backcross animals was used as the QT of interest. The percent immunosuppression (expressed as a fraction) was arcsine square root transformed. The between-experiment variation was corrected by expressing the results as *Z*-score based on the mean and standard deviation of each experiment.

#### Statistical Significance Assessments

For the GAW15 Data set, we used all 100 replicates to obtain KWII and PAI values and the corresponding 95% confidence intervals for each combination of variables. The number of potential tests for all one-way and two-way combinations was 42,232,645 (consisting of 9,190 one-way and 42,223,455 two-way) each for the IgM and Anti-CCP QT in the GAW15 data set.

For the high density lipoprotein and atherosclerosis and ultra-violet light induced immunosuppression datasets and the first replicate of the GAW15 Data set, the null distribution of the KWII of each combination was obtained from 5000 random permutations of the QT values. The permutations for each combination were conducted independently. The raw *p*-value of the KWII of a combination was ascertained as the fraction of the permuted KWII values that were equal to or greater than the observed KWII value. The same permutation approach was employed for the HDL and atherosclerosis data sets and for the first data replicate in the GAW15 data set to determine the time required for permutations; the top 25 one-way and top 25 two-way combinations were analyzed.

The number of potential tests for all one-way and two-way combinations was 6,216 (111 one-way plus 6,105 two-way) each for the HDL and atherosclerosis data sets and 2,145 (65 one-way plus 2,080 two-way) for UV light-induced immunosuppression data set.

### Similarity and Differences with the Restricted Partitioning Method

The Restricted Partitioning method [33] (RPM) code was provided by Dr. Culverhouse. It was used to analyze the HDL data set [25] previously described. The α-value was set to 0.01.

## Results

### Performance of K-Way Interaction Information (KWII) and Phenotype-Associated Information (PAI) on Simulated Data

In these experiments, our goal was to compare the effectiveness of K-way interaction information (KWII) and phenotype-associated information (PAI) on simulated data with known patterns of interactions. These Simulated Data Sets were intentionally kept simple so that the heuristics for interpreting the KWII and PAI could be identified.

#### Simulated Data Set 1, Simple Epistasis

Figures 2B and 2C show the KWII and PAI, respectively, for each combination of interactions between the phenotype and all possible SNP combinations. We refer to these graphs as KWII and PAI spectra.

The combinations are shown on the *y*-axis; e.g., 1, 2, *P *indicates that variables SNP 1, SNP 2 and the QT variable, *P*, are used in calculating *k*-way (*k *= 3) interaction. The KWII spectrum contains two dominant peaks corresponding to the {*1*, *P*} and {*2*, *P*} combinations indicating that SNPs 1 and 2 contribute significantly to the quantitative trait variable *P*. The permutation-based *p*-values of the KWII for both combinations was *p *< 0.0002. Qualitatively, the stepwise structure of the PAI spectra contrasts with the peak-like structure of the KWII spectra. The presence of a higher peak for the {*1*, *2*, *P*} combination compared to the {*1*, *P*} and {*2*, *P*} combinations indicates a dependence of SNP 1 on SNP 2. Thus, the PAI detects the dependence between SNPs 1 and 2 and provides information that complemented the KWII. The variables *not *involved in interactions (SNPs 4, 5 and 6) can be more easily identified from the PAI spectra because the PAI values for combinations containing these independent variables are the lowest compared to combinations containing interacting variables. None of *p*-values of the KWII for variables not involved in interactions were significant (all *p*-values > 0.05).

Figure 2D shows the dependence of KWII on the effect size. In Figure 2D, the KWII of three representative combinations: the 1-variable containing {*1*, *P*} and {2, *P*} combinations and the 2-variable containing {*1*, *2*, *P*} combination are shown. The KWII values of all three combinations increased with increasing effect size. We also computed the 95^th ^and 5^th ^percentiles of the KWII each peak to assess variability due to sampling; in some of the cases, the standard deviation was quite low and obscured by the symbols used for graphing. These results demonstrate that the KWII spectra convey information on the types of interaction present and also their effect size.

We incorporated pairwise LD between the SNP 1 and SNP 3 pair and between the SNP 2 and SNP 4 pair. The *R*^2 ^values for the LD for both SNP pairs were set equal and the value was increased from 0 to 0.95. The effects of LD on the PAI for two combinations, {*1*, *3*, *P*} and {*2*, *4*, *P*} are summarized in Figure 2E. The results demonstrate that the PAI is independent of LD and the interaction information related to the QT is retained.

Figure 2F shows the power of KWII at a sample size of 5000 per group for various effect sizes for {*1*, *P*}, {*2*, *P*} and {*1*, *2*, *P*} combinations. The power of the KWII was greater than 0.99 for all three combinations at an effect size of 0.5 or greater.

#### Simulated Data Set 2

This Simulated Data Set is relatively complex and contains a combination of interactions among and between the environmental and genetic variables. In addition, 1-variable, 2-variable and 3-variable containing GGI and GEI interactions with the QT variable, *C*, are incorporated in the model.

The one-variable containing peaks in the KWII spectrum correctly identified the critical roles of *E1*, *E2*, *SNP(1) *and *SNP(2) *variables (all *p*-values < 0.0002) in the underlying model and the two-variable containing {*E1*, *E2*, *P*} and {*1*, *2*, *P*} interactions (*p *< 0.0002 and *p *= 0.015, respectively) were also identified. Furthermore, the three variable containing {*E2*, *1*, *2*, *P*} combination (*p *< 0.0002) was the three-variable combination with highest the KWII values.

The PAI spectrum complements the KWII spectrum. For 1-variable containing interactions the values of KWII and PAI are identical and equivalent to mutual information. However, the most notable characteristic of the PAI spectrum is its discrete step-like visual appearance. Each interacting variable in the data set contributes approximately one single unit step. The height of the PAI peak increases whenever an informative variable is added to the variable list and is unchanged when a non-interacting variable is added. The lowest values of PAI correspond to the subsets containing only non-interacting variables such as {*3*, *4*, *P*}, {*3, 5, P*}. By identifying the largest subset with low PAI values, the spectrum can be used to eliminate non-interacting variables.

Figure 3D shows the effect size dependence of KWII for three representative informative 1, 2, or 3-variable containing combinations, {*E2*, *P*}, {*E1*, *E2*, *P*} and {*E2, 1*, *2*, *P*}, from Figure 3B. As in Simulated Data Set 1, the KWII for each combination increased by almost an order of magnitude for the 5-fold increase in effect size. The 95^th ^and 5^th ^percentiles of KWII were also computed and variability for Simulated Data Set 2 was qualitatively greater than for Simulated Data Set 1 possibly because of the complexity and number of the underlying GEI. The coefficients of variation of the KWII values decreased with increasing effect size.

The effects of LD (for *R*^2 ^ranging from 0 to 0.95) on the PAI for two combinations, {*1*, *3*, *P*} and {*2*, *4*, *P*} for effect size of 0.5 are summarized in Figure 3E. Again, the results demonstrate that the PAI is robust and independent of LD.

Figure 3F shows the power of KWII at a sample size of 5000 for various effect sizes for three representative informative 1, 2, or 3-variable containing combinations: {*E2*, *P*}, {*E1*, *E2*, *P*} and {*E2, 1*, *2*, *P*}. The KWII had a power of 0.89 at an effect size of 0.25 for the {*E2*, *P*} peak. At an effect size of 0.5, the power for the {*E1*, *E2*, *P*} and {*E2, 1*, *2*, *P*} peaks were each 0.98 or greater.

#### Simulated Data Set 3

This Simulated Data Set investigated a two-locus model of pure epistasis. Pure epistasis models lack QT variation attributable to single-loci and require interactions between different loci to explain the variation [23].

As would be predicted from the simulation framework, the KWII spectra for the two-locus (Figure 4A) pure epistasis interaction model did not contain any peaks corresponding to single-SNP combinations or to uninformative SNP combinations (all *p*-values > 0.05). The KWII spectrum for the two-locus model contained a prominent peak (*p *< 0.0002) corresponding to the informative two-SNP combination {*1*, *2*, *P*}. This suggests that the interactions identified with the KWII are qualitatively concordant with epistatic interactions. Figure 4B shows that the power of detecting the epistatic interaction based on the KWII of {*1*, *2*, *P*} combination is satisfactory.

Although the KWII correctly detects epistatic interactions, CHORUS is not capable of identifying these interactions because it employs a marginal effect search strategy. The models used in this Simulated Data Set exhibit pure epistasis only when the allele frequencies of the interacting SNPs are 0.5. We hypothesized that differences in allele frequencies between the interacting loci would cause deviations from pure epistasis and result in the appearance of KWII peaks for lower-order combinations. Figure 4C shows the dependence of the KWII on the allele frequency at SNP(2) in the two-locus model of epistasis. When the allele frequencies of both loci are equal, the KWII values of the one-variable combinations are nearly zero as expected. However, the KWII value of the {*1*, *P*} combination increases with deviations from equality of allele frequency. The KWII value for the {*1*, *2*, *P*} combination is maximal when the allele frequencies are equal and decreases with deviations from equality.

Taken together, these Simulated Data Sets demonstrate that the KWII and PAI spectra are capable of visually summarizing a diverse range of gene-environment interaction phenomena.

### Performance of PAI-Based CHORUS Algorithm on Public Domain Sets

#### 10K GAW15 Dataset

The underlying GGI and GEI in the simulations for this data set models the interaction of nine loci: C, DR and D on chromosome 6, A on chromosome 16, B on chromosome 8, E on chromosome 18, F on chromosome 11, G and H on chromosome 9 (see Table 1 for a summary) [24]. The associations between the individual loci and RA affection status and the Anti-CCP and IgM that were built into the data set by Miller et al. [24] are summarized in Table 1. The Anti-CCP and IgM were QT and the focus for this report; the associations corresponding both QT are highlighted in bold in Table 1. Note that the Anti-CCP and IgM measures are defined for the cases only. Although phase information was provided, we treated the data as genotype data for the CHORUS analysis.

The GAW15 data set contained 100 replicates from repetitions of the simulation procedure [24] and the availability of these replicates enabled us to compute the 95% confidence interval for the KWII of each combination.

**Figure 5:**
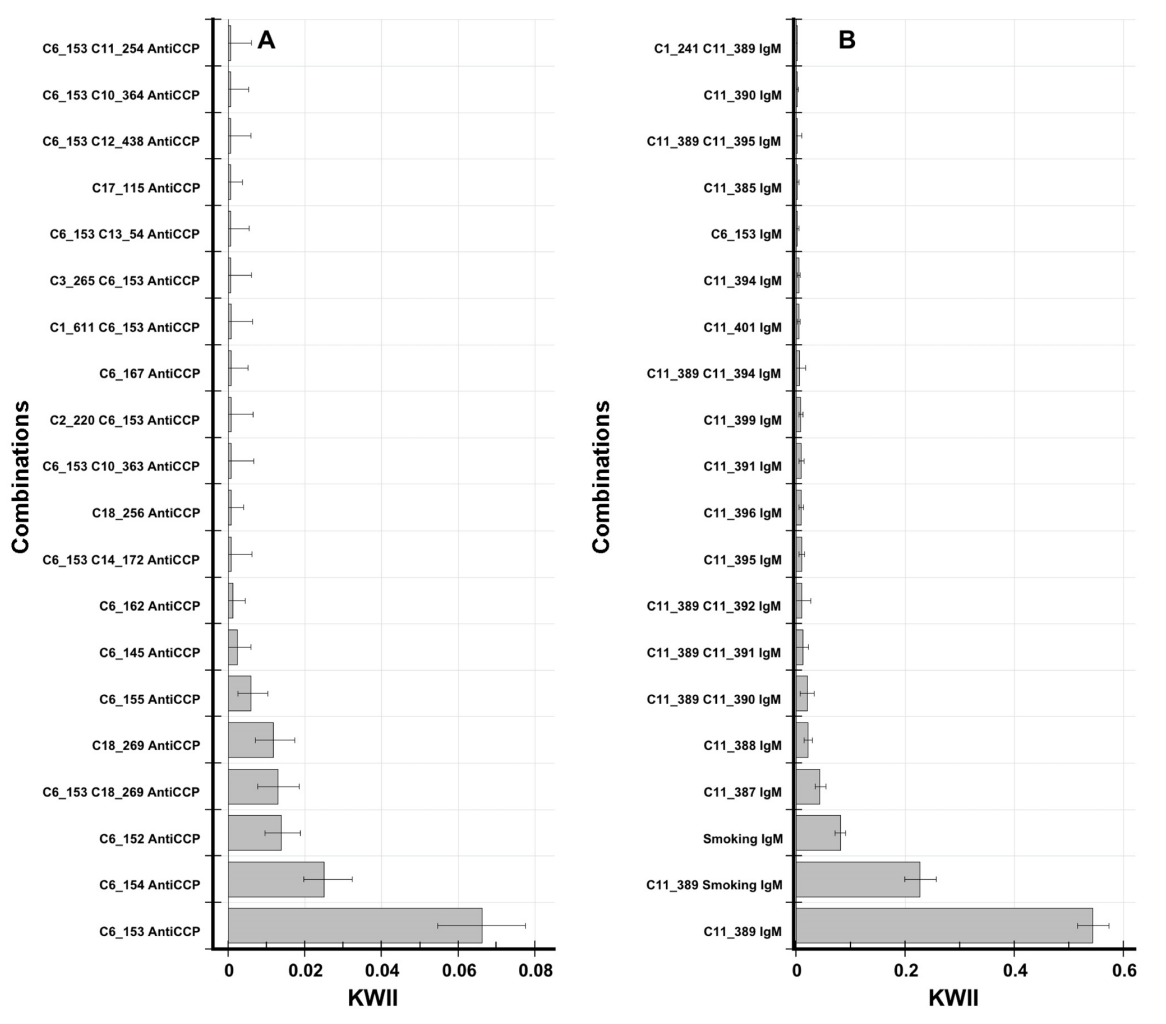
**The KWII spectra for one-variable and two- variable containing combinations for the "10K GAW15 data set" with Anti-CCP antibody status (Figure 5A) and IgM status (Figure 5B) as the quantitative traits of interest are shown**. The variable combinations are indicated on the *y*-axis; the chromosome number and the SNP identifiers are provided for SNPs. The bars represent mean values and the upper and lower error bars are the 95^th ^and 5^th ^percentiles of KWII values, respectively.

Figure 5A presents the KWII values for various combinations with Anti-CCP as the QT of interest. The peaks with the highest KWII values (Figure 5A) enabled the identification of the following loci and covariates associated with Anti-CCP: loci C, DR (chromosome 6), locus E (chromosome 18), Age and IgM. The strongest contributions to the Anti-CCP in simulations were from Locus E and DR; Locus E affects Anti-CCP by controlling which DR genotypes place a subject in the Anti-CCP group with high mean values [24]. Figure 5A demonstrates that the three highest KWII correspond to the interaction between the DR locus (SNPs C6_152-C6_155) and Anti-CCP (all *p*-values < 0.0002); the next two peaks, {*C18_269, Anti-CCP*} and {*C6_153, C18_269, Anti-CCP*}, correspond to the interactions of Locus E alone and the Locus E, DR combination with Anti-CCP (both *p *< 0.0002).

Figure 5B presents the KWII values for various combinations with IgM as the QT of interest. The peaks with the highest KWII values enabled the identification of the following loci and covariates associated with IgM: loci C and DR (chromosome 6), locus E (chromosome 18), Smoking: the highest KWII peaks corresponded to {*C11_389, IgM*}, {*C11_389, Smoking, IgM*} and {*Smoking, IgM*} in Figure 5B (all *p*-values < 0.0002). It is important to note that the KWII for each of these combinations does not contain redundant information. Thus, the significant peaks for the {*C11_389, Smoking, IgM*} and {*Smoking, IgM*} indicate that *Smoking *alone is IgM-associated but also contributes to IgM synergistically in association *C 11_389*. Furthermore, the changes in the peak height should not be interpreted to imply any protective role for *Smoking*.

The KWII spectrum derived from CHORUS identified all key covariates associated with the IgM and Anti-CCP without the use of haplotype-phase information or the parent-child transmission information contained in the pedigree structures.

#### HDL and Atherosclerosis Dataset

**Figure 6:**
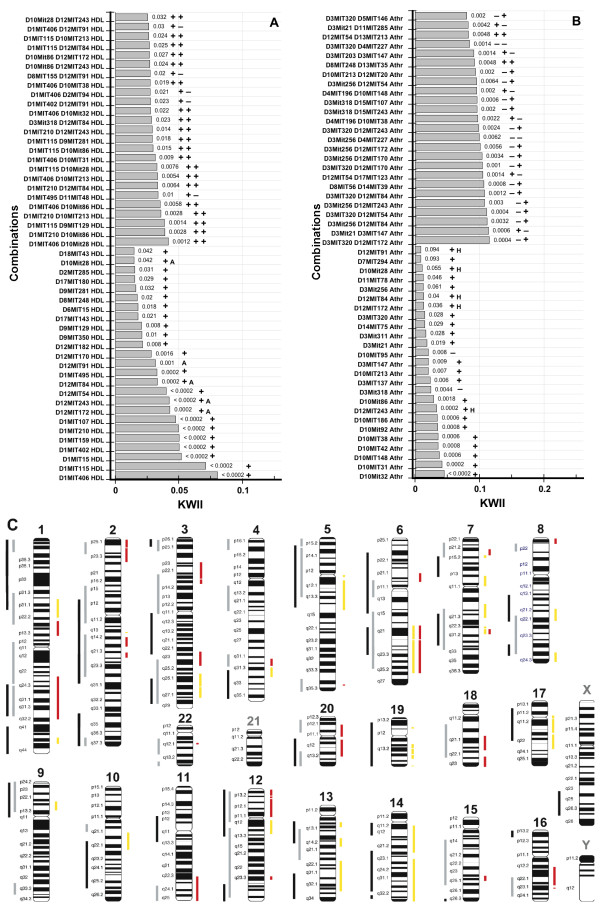
**The KWII spectra for HDL (Figure 6A) and atherosclerotic lesion length (denoted by the variable *Athr *on the *y*-axis of Figure 6B)**. The *p*-values for each combination are shown against the bars. The plus symbol indicates that there is a human QTL reported for the orthologous region corresponding to the mouse marker and the minus symbol indicates that a human QTL has not been reported. For the two-variable combinations, the plus and minus symbols are placed in the same order as the *y*-axis labels. The A and H indicate if the marker is present in both the HDL and Atherosclerosis spectra. Figure 6C is a haploid human karyotype ideogram. To the right of each chromosome, the human orthologs of the HDL (red bars) and atherosclerotic streak length (yellow bars) associated mouse markers identified in our study are shown. To the left of each chromosome the human QTL for HDL (gray bars) and atherosclerotic disease (black bars) are shown. Chromosomes 21, X and Y contained no overlapping orthologs and their labels are shown in gray.

For HDL susceptibility, Ishimori et al. [25] identified five significant single locus effects on plasma HDL concentrations: *D1Mit159*, *D1Mit406, D2Mit285*,,, *D9Mit129 *and *D12Mit172*; one locus, D8Mit248, was suggestive of linkage. Using pair-wise scans, they also found two gene interactions involving *D1Mit406*: {*D1Mit159*, *D1Mit406*} and {*D1Mit406*, *D2Mit285*} [25]. From the CHORUS analysis (Figure 6A), three loci: *D1Mit406*, *D12Mit172 *and *D1Mit159 *were significant even after a conservative Bonferroni correction for multiple testing and all six loci were among the top 25 single SNP combinations.

However, our two SNP results did not agree with those of Ishimori et al. [25]. The 2-SNP containing combination {*D1Mit159*, *D1Mit406*} was found to have negative KWII indicating redundancy most likely because of the strong single SNP effect of each SNP (*p *< 0.0002). Also the combination {*D1Mit406*, *D2Mit285*} was not found among the 2-SNP combinations with the highest KWII values; both *D1Mit406 *(*p *< 0.0002) and *D2Mit285 *(*p *= 0.031) were individually associated with HDL (Figure 6A). Interestingly we did detect interactions between *D1MIT406 *locus on Chromosome 2 and markers identified by Ishimori et al. [25] for atherosclerotic aortic fatty streak lesion size, e.g., {*D1MIT406*, *D10MIT213*} and {*D1MIT406*, *D10MIT31*}. The mouse markers associated with both HDL and Atherosclerosis QTs in our analysis are annotated (with an *A *in Figure 6A and an *H *in Figure 6B).

For atherosclerotic aortic fatty streak lesion size, Ishimori et al. identified five loci that accounted for 35% of the variance in multiple regression analysis [25]. Of these, *D10Mit31 *was a significant QTL on chromosome 10 wherein a dominant B6 allele conferred atherosclerosis resistance. *D12Mit243 *was suggestive as a single QTL and was significant in gene interaction with D11Mit333. Another interaction was found between the loci *D10Mit213 *and *D12Mit7*. In the CHORUS analysis (Figure 6B), we were able to identify three of the five susceptibility loci but as single locus interactions. *D10Mit32 *was found to have the highest KWII value and was in strong LD with *D10Mit31*. *D12Mit243 *and *D10Mit213 *were also found to have significant single locus effect on atherosclerotic aortic fatty streak lesion size.

Figures 6A-C summarize the results from mouse-human comparative genomics analysis to critically evaluate the biological significance of the markers identified by our method. The plus and minus symbols in Figure 6A and 6B indicate whether or not the orthologs corresponding to a ± 16 Mbp region of the mouse markers identified by the CHORUS method overlapped with human QTLs for HDL and atherosclerotic disease. For HDL (Figure 6A), all 25 (100%) of the single marker combinations with the highest KWII values were concordant with the human QTL for HDL. Among the two-marker combinations, 20 of 25 were concordant on both markers and 5 of 25 were concordant for one of the two markers. For atherosclerotic disease (Figure 6B), 23 of 25 (92%) of the single marker combinations with the highest KWII values were concordant with the human QTL for atherosclerotic disease and among the two-marker combinations, 23 of 25 were concordant on at least one of the two markers. The high degree of concordance with human QTL demonstrates usefulness of our approach in humans. In Figure 6C, we have represented the human QTL against the backdrop of a human haploid karyotype ideogram.

The underlying genetic etiology of HDL and atherosclerotic diseases appear to be shared. Human atherosclerotic diseases include myocardial infarction, coronary artery disease, acute coronary syndrome (acute myocardial infarction and unstable angina), carotid artery intimal-medial thickness disease, coronary artery calcium, peripheral arterial occlusive disease and stroke [27,29]. To date, atherosclerotic disease linkage studies have been conducted in 19 human populations and 40 atherosclerotic disease-regulating QTL have been identified. Two meta-analysis have confirmed regions on 2q and 3q [34] and (1p, 5p, 12q, 13q) while four new linkage regions (6p, 2 on 8q, 14p) not found in original studies have been revealed [35]. Previously, 31 of the 40 human QTL were found to be concordant with mouse QTL [26], suggesting that identifying the genes underlying the mouse QTL could potentially facilitate identification of the genes underlying the human QTLs. To date, only 3 putative disease genes from these QTLs have been found for atherosclerotic disease [36,37]. The comparative genomics approach was used to determine if orthologs of mouse markers identified by CHORUS overlapped with the 40 human QTLs. As summarized in the human haploid karyotype ideogram in Figure 6C, we successfully identified orthologous regions of the mouse markers identified by CHORUS overlapping with the substantive majority of the human 40 QTL demonstrating: i) high concordance between human QTL and the mouse markers identified by CHORUS, and ii) that CHORUS in conjunction with comparative genomics can enable identification of more limited, narrower regions of overlap. These narrow regions may merit fine mapping studies. Furthermore several regions had QTLs for both HDL and Atherosclerosis, a biologically plausible result not previously found.

The 7q22 region (Figure 6C) exhibits a small region of overlap between HDL and Atherosclerosis phenotypes in humans. In 2002, a combined analysis of genome scans for obesity was undertaken using the interim results from the National Heart, Lung, and Blood Institute Family Blood Pressure Program. The sample represents the largest single collection of genome-wide scan data that has been analyzed for obesity and provide a test of the reproducibility of linkage analysis for a complex phenotype. Body mass index (BMI) was used as the measure of adiposity and this analysis confirmed linkage to the 7q22.2 region [38]. The result from a hypothesis free analysis clearly indicate that this is a viable region for fine mapping either heart disease endophenotypes, such as body mass index or Atherosclerotic phenotypes previously discussed. Further interrogation of this region has shown that it contains the gene visfatin (Gene Symbol PBEF1; also called nicotinamide phosphoribosyltransferase or Nampt and pre-B-cell colony enhancing factor or PBEF1) an adipokine found in abundance in visceral fat. This gene has been shown to lower plasma glucose in both humans and mice [39]. More specifically, there is evidence that visfatin levels are correlated with HDL-C and apolipoprotein A1 in Asian Indians [40] and recently evidence of allelic association with SNPs in visfatin and plasma levels has been shown in a Chinese population [41]. The results demonstrate that our method is capable of successfully identifying the same regions at the genome wide level as have been found and confirmed. Although follow up candidate gene studies have mapped visfatin, the power of our approach lies in the ability to map and narrow regions of GEI when doing the initial genome wide scan if given clinical and demographic variables.

#### UV-Induced Immunosuppression Data Set

Clemens et al. identified four quantitative trait loci (QTLs) with significant main effects for UV-induced immunosuppression on Chromosomes 1, 6, 10 and 17 [32]. The locus on Chromosome 1 had interactions with two other loci on chromosomes 14 and 19 [32].

**Figure 7:**
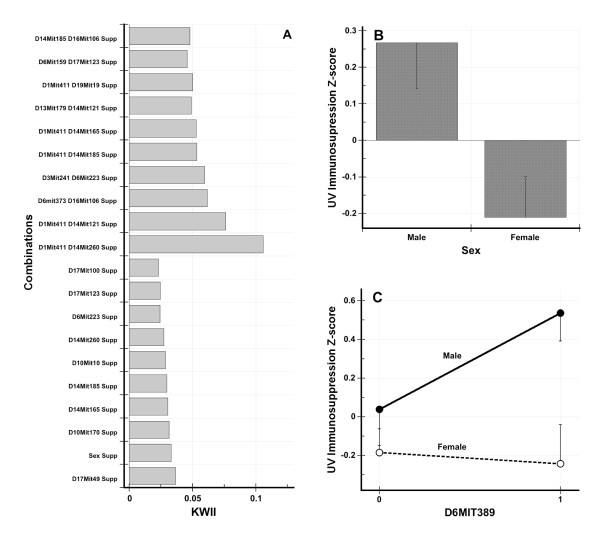
**The KWII spectrum for the UV-induced immunosuppression data set is summarized in Figure 7A**. The combinations are shown on the *y*-axis. The UV-induced immunosuppression denoted by the variable *Supp *on the *y*-axis. Figure 7B shows the UV-induced immunosuppression *Z*-score in males and females whereas Figure 7C shows the UV-induced immunosuppression *Z*-scores for the two genotypes of *D6MIT89 *in males and females. The error bars represent standard error of the mean.

The KWII spectrum for the UV-induced immunosuppression data set is summarized in Figure 7A. CHORUS identified several of the same single locus and two locus interactions found by Clemens et al. *D10Mit170 *on chromosome 10 and *D17Mit49 *on chromosome 17, the two most significant single loci found by Clemens et al. [32], were also the two single loci with the highest KWII values identified by CHORUS. The two marker interactions *{D1Mit411, D19Mit19}, {D1Mit411, D14Mit185} *and *{D1Mit411, D14Mit260} *reported to be significant by Clemens et al., were among the top ten highest KWII scores; *{D1Mit411, D14Mit260} *was the two-variable interaction with the highest KWII value.

In Table 2, we compare the *F*-interaction values that were obtained by the Clemens et al. [32] for 11 different marker pairs from regression analyses to their corresponding KWII values. The KWII and *F*-interaction values (Spearman rank correlation *ρ *= 0.86, *p *= 0.001) were highly correlated. However, our analysis identified a potentially significant effect of *Sex *on UV-induced immunosuppression whereas Clemens et al. [32] reported the absence of significant associations. Specifically, *Sex *had the second highest KWII value and the *D6Mit389 *marker was found to interact with *Sex*. To more thoroughly assess whether the underlying data shows evidence of the interaction identified by the KWII, we plotted the UV-induced immunosuppression results for males and females (Figure 7B) and for each value of *D6Mit389 *(Figure 7C). In Figure 7C, for a *D6Mit389 *genotype value of 1, we observed prominent differences between males and females. In a GLM analysis (*F *= 4.2, *p *= 0.008 for overall model) with terms for *Sex*, *D6Mit389 *and an interaction between *Sex *and *D6Mit389*, we found *Sex *to be significant (*F *= 9.1, *p *= 0.003) and a trend toward significance for the interaction term (*F *= 2.8, *p *= 0.097). Similar sex differences were reported in an independent study by Noonan et al. [42] These authors found a significant sex difference in backcross mice derived from BALB/c and C57BL/6 inbred strains: male mice were more susceptible to UV-induced immunosuppression [42]. The incidence and mortality of skin cancer is higher in men compared to women and a recent human study employing the Mantoux reaction found that men were immunosuppressed by UV doses three times lower than those for women [43].

**Table 2:** Summary of the analysis of marker pairs by Clemens et al. [25,32]

Marker Pair	F-interaction	P-value	KWII	KWII p-value
*D6Mit389-D10Mit170*	2.63	0.11	-0.0017	0.76
*D10Mit170-D17Mit49*	0	0.97	-0.0079	> 0.99
*D10Mit170-D17Mit123*	0.01	0.91	0.0047	0.38
*D10Mit170-D17Mit187*	0.04	0.85	0.0005	0.59
*D10Mit170-D17Mit100*	0.01	0.91	-0.0078	> 0.99
*D10Mit170-D14Mit185*	5.38	0.02	0.0087	0.22
*D10Mit170-D14Mit165*	3.78	0.055	0.0031	0.43
*D1Mit411-D14Mit260*	12.9	0.00053	0.10	< 0.0002
*D1Mit411-D14Mit185*	12.3	0.00069	0.048	0.004
*D1Mit411-D19Mit19*	12.0	0.00079	0.042	0.006
*D1Mit411-D17Mit49*	0.98	0.32	0.0020	0.48

### Similarity and Differences with the Restricted Partitioning Method

We assessed the similarities and the differences between the results from CHORUS to the Restricted Partitioning Method (RPM), a competing method for GEI analysis of QT, using the HDL data set [25].

RPM identified 21 one-variable containing combinations as significantly associated with HDL. Twenty of these were among the top 20 one-variable combinations with the highest KWII values. The concordance on the one-variable combinations is reassuring given that RPM and CHORUS use different approaches to detect between-group differences. RPM identified 1905 two-variable containing combinations with *p*-values of 0.05 or less; of these 599 combinations had *p*-values < 0.0002. All twenty of the top 20 two-variable combinations with the highest KWII values were identified as significant by RPM.

**Figure 8:**
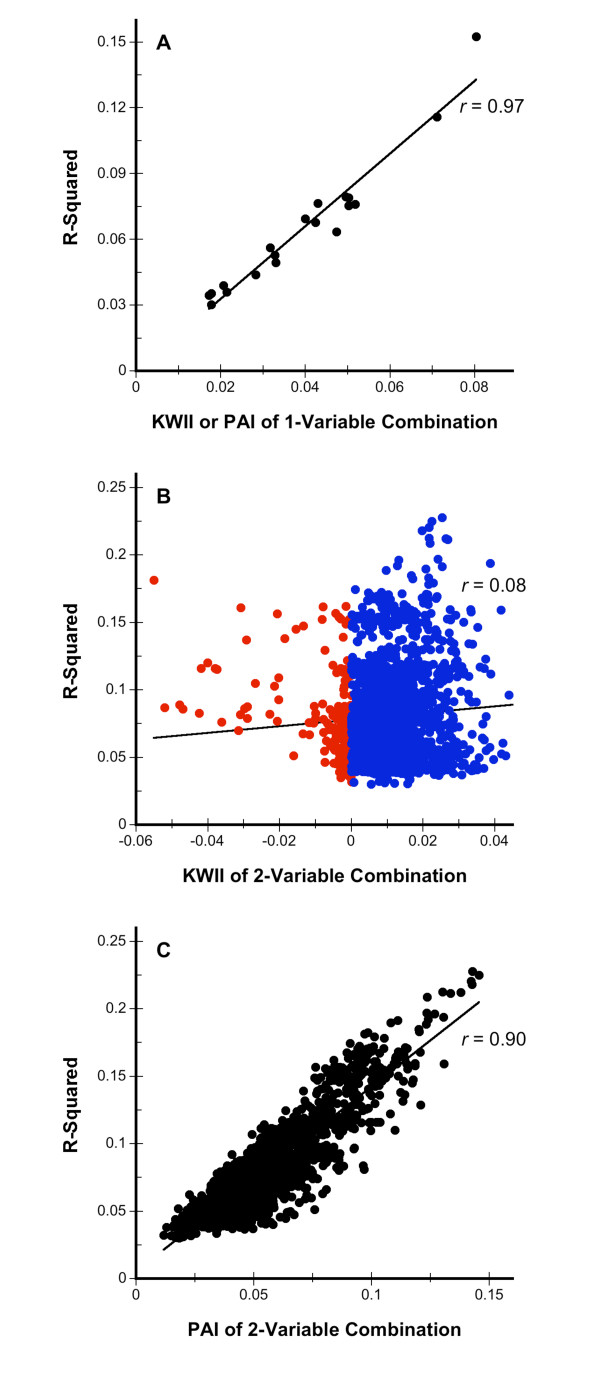
**Associations between the KWII and PAI values with *R*^2 ^values from the Restricted Partitioning method**. Figure 8A shows the KWII and PAI results for the one-variable containing combinations (the PAI and KWII are equal for one-variable combinations). The KWII and PAI for two-variable combinations are shown in Figure 8B and 8C, respectively. The red and blue circles in Figure 8B represent combinations with negative and non-negative KWII values, respectively. The linear regression line and its Pearson correlation coefficient *r *are shown.

To assess the reasons underlying the qualitative concordance and distinctive differences between the RPM and CHORUS methods, we plotted the KWII and PAI values for one-variable and two-variable combinations found significant by RPM against the *R*^2 ^value in the RPM output. For the one-variable combinations (Figure 8A), the KWII and PAI values are equal and were found to be correlated with the *R*^2 ^with a Pearson correlation coefficient *r *= 0.97. This explains in part the high degree of concordance between the two methods for one-variable combinations. However, for the two-variable combinations, the KWII (Figure 8B) was not linearly correlated with the *R*^2^; the Pearson correlation coefficient was only *r *= 0.08. Furthermore, numerous combinations identified as significant by RPM had negative KWII values indicative of redundancy, that are shown in red in Figure 8B. The PAI however, was linearly correlated with *R*^2 ^with Pearson correlation coefficient of *r *= 0.90. These results suggest that the RPM algorithm retains redundancies that are not present in CHORUS. Thus, the similarities between the RPM and CHORUS methods can be attributed to the correlations between the *R*^2 ^values of RPM and the PAI, whereas the differences are attributable to the lack of correlation between the *R*^2 ^values of RPM and the KWII.

### Computational Complexity

The CHORUS search algorithm is computationally much more efficient than the exhaustive search to compute KWII for all possible SNP combinations, which requires exponential time.

Let *m *denote the sample size. In the CHORUS search algorithm, lines 3-5 take *O*(*n × m*^2^), line 6 require *O*(*θ × n*) time whereas lines 7-16 takes *O*(*τ × θ × n × m*^2^) time and lines 19-25 takes *O*(2^τ+1 ^× *θ *× *m*^2^) time. In GEI analysis of QT, the range of *τ *values of interest is small because of sample size constraints, which limits the computational complexity from becoming exponentially large. When ω permutations are conducted for every combination identified by CHORUS, the computations require *O*(2^*τ*+1 ^× *θ *× *m*^2 ^× ω) time.

The GAW15 data set was analyzed on a 3.2 GHz Intel Xeon computer with Irwindale processors with 4 Gb of main memory. The maximum running time for single replicate of the GAW15 data set was 3.6 hours with *τ *= 2 and θ = 50 excluding permutations. The permutation analysis of the first replicate of the GAW15 data set, which was conducted for the 25 one-way and 25 two-way combinations with the highest KWII values with 5000 permutations per combination, required 5 hours and 25 minutes. The HDL and atherosclerosis data sets were analyzed using a Dell Laptop with 2 GHz dual core Intel processor with 2 Gb of memory. The time required was approximately 8 seconds for both data sets excluding permutations. The permutation analysis (5000 permutations per combination) for the top 25 one-way and top 25 two-way combinations required 14 minutes for the HDL data set and 16 minutes for the atherosclerosis data set.

## Discussion

In this report, we have presented results on an information theoretic approach for GEI analysis of QT that uses two complementary information-theoretic metrics, the KWII and the PAI. The dependence of these metrics on biological and study design variables was systematically investigated with controlled numerical experiments. We analyzed the GAW15 data set, which was generated by Miller et al. [24] from a complex simulation based on rheumatoid arthritis data and two GGI data sets generated from QTL mapping studies of HDL levels/atherosclerotic lesion size [25] and UV-induced immunosuppression [32].

The current method assumes that the QT of interest is normally distributed within each strata of the gene-environmental variable combination. The normal distribution is a common assumption in parametric statistics and derives its importance from the central limit theorem. From the information theoretic standpoint, the normal distribution *N*(*μ*, *σ*) has maximum entropy among all real-valued distributions with specified mean *μ *and standard deviation *σ*. Therefore, if only the mean and standard deviation of a distribution are known, it is often reasonable to assume that the distribution is normal. As we demonstrated, a variety of data transformations such as log-transformation, arcsine transformation and others can sometimes be used to obtain normal distributions in some cases when the underlying variable is non-normally distributed. Although the normality requirements for each genotype-environment stratum could be considered a strong assumption, it is possible to deal with mixed distributions or empirically estimate the distribution of the QT in each stratum, e.g., with Parzen windows [44], and use the information theoretic framework and CHORUS in consistent and analogous manner.

In the case of a normal distribution, the entropy expression contains only the variance. As a result, the approach conveys the impression of being driven by the variance. We have not addressed standard deviation estimation issues in detail here because our primary focus was to determine whether the underlying method was capable of identifying GEI. Greenwood and Sandomire demonstrate that at a sample size of 25, a standard deviation estimate is within ± 10% error half the time [45].

Derivations of information theoretic metrics in terms of statistical parameters such as variance result in analytical expressions that are difficult to interpret intuitively. Set theoretic approaches provide more interpretability because they can account for addition and subtraction of entropies. Unlike the variance or second moment, which measures dispersion around the mean, entropy depends on parameters other than just the second moment, e.g., the shape and scale parameters of the distribution of interest. One advantage of the information-theoretic method is that it is capable of handling mixtures wherein the strata have different distributions.

The KWII definition of an interaction has a strong theoretical foundation from information theory and the statistical significance of the KWII can be assessed using permutation-based methods. Because the distribution of the KWII and PAI of higher order combinations has not been characterized, we used independent replicates for the three Simulated Data Sets and GAW15 data set to directly obtain confidence intervals as well as empirical information on the distribution of the KWII and PAI values. In the case of the GAW15 data set our approach enabled use of the entire data set. This approach is not feasible for real data and permutations are necessary to assess statistical significance via *p*-values. Permutations however, provide information on the null distribution.

As indicated in Methods, the KWII-based definition of interaction yields results that difficult to interpret for completely redundant variables because in the presence of an even number of completely redundant variables, the KWII is positive. This quandary can be addressed by retaining only one representative variable from every group of completely redundant variables in a pre-processing step prior to analysis. However, the PAI does not change when a completely redundant variable is added to combinations containing odd or even number of completely redundant variables. Because the CHORUS search of combinatorial space is directed towards combinations that increase PAI, our approach is less susceptible to identifying combinations comprised of variables that are completely redundant with each other.

The CHORUS algorithm however, is a heuristic method. CHORUS uses a search strategy rather than a dimensionality reduction approach and is capable of conducting efficient search of the large combinatorial space because of the unique nature of the PAI metric, which allows for greedy search identification of the most promising combinations by utilizing the marginal effects. As a consequence however, CHORUS is not capable of detecting pure epistasis. It is possible to develop a "two-locus" variation of CHORUS that utilizes KWII from all one-variable and two-variable combinations.

For Simulated Data Set 3, we adopted the overall structure, key assumptions and numerical values from previous work on pure epistasis in case-control data by Culverhouse [23]. Our simulations assumed Hardy-Weinberg equilibrium and MAF of 0.5 at the interacting SNPs, *SNP(1) *and *SNP(2)*. The frequency of each genotype in our sample was representative of the corresponding population frequencies. The QT value for each subject was a random variate drawn from one of two normal distributions *N*(*μ*_1_, *σ*_1_) or *N*(*μ*_0_, *σ*_0_). The probability of drawing the QT random variate from *N*(*μ*_1_, *σ*_1_) was specified for each combination of *SNP(1) *and *SNP(2) *genotypes Equation 1 obtained from [23]. The probability of drawing from *N*(*μ*_0_, *σ*_0_) was defined by the complement of the probabilities in Equation 1. These assumptions result in a form of QT epistasis because the two distributions *N*(*μ*_1_, *σ*_1_) and *N*(*μ*_0_, *σ*_0_) can be considered cases and controls and the model can be viewed as a binary trait to which normally distributed noise has been added.

CHORUS can be considered complementary to dimensionality reduction methods such as combinatorial partitioning method (CPM), multi-factor dimensionality reduction (MDR) and restricted partitioning method (RPM), which are computationally more burdensome but are sensitive to pure epistasis interactions. The CPM approach is capable of identifying multilocus genotypes capable of predicting QT levels [46]. The multi-factor dimensionality reduction (MDR) method is applicable to binary phenotypes and uses constructive induction to reduce the dimensionality of the multi-locus genotype systematically by pooling into high and low risk groups [3,47-50]. The CPM is computationally very intensive and Culverhouse et al. advocated the RPM [33], which is applicable to both binary phenotypes and QT. Although RPM and MDR are computationally more efficient than the CPM, significant computational effort is required for datasets from genomewide association studies, which can contain tens of thousands to millions of predictor variables. The generalized MDR (GMDR) method handles both discrete phenotypes and continuous traits in population-based study designs and employs the generalized linear model (GLM) framework for scoring in conjunction with MDR for dimensionality reduction [51].

Unlike exhaustive search algorithms, which can identify the global minimum, all heuristic approaches are potentially vulnerable to entrapment in local minima. CHORUS can be modified with established methods such as simulated annealing to reduce this risk. Within the current CHORUS framework, the input parameter θ, which determines the number of combinations retained at each stage of the algorithm, can also be a determinant of power: if too few combinations are retained at the initial stages of the search, the risk of missing key higher-order interactions with intermediate levels of marginal effects is increased. However, increases in θ increase the computational cost. In principle, the computational effort depends exponentially on the input parameter τ, which determines the order of combinations. In practice however, the value of τ is constrained by sample size because the genotype contingency tables for combinations rapidly become sparse and contain numerous empty cells when the order of the combinations increases. Although biological pathways are complex, sequentially ordered actions of protein-protein interactions and enzymatic chemical reactions are frequently involved [52]. Such sequential interactions typically involve only a small subset of molecules in the pathway. The order of resulting statistical interactions may be limited as a consequence [15].

There are some fundamental differences and unique advantages to CHORUS compared to the widely used GMDR approach. The metric used by GMDR is based on the GLM, a commonly used and versatile statistical analysis method, and is combined with dimensionality reduction method of MDR. For QTL analysis, GMDR analyzes the QT and covariates first to obtain the GLM score statistic and in a second stage, the interactions of GLM score statistics with the genetic and environmental variables are determined. In contrast, the CHORUS approach analyzes the underlying interactions between the QT of interest and all variables including covariates simultaneously. Another advantage with CHORUS is that it is capable of handling cases-only study designs that are useful for studying the genetic and environmental determinants of important QT such as body weight, height and lifespan. The statistical GLM framework enables GMDR to handle covariates whose distribution follows any of the exponential family distributions (normal, Poisson or Bernoulli distributions) but a limitation of CHORUS that we are working to overcome is that it cannot handle continuous covariates. Continuous covariates can be used after discretization, however. Another notable advantage of CHORUS is that it can be applied to very large data sets. We were able to analyze the 100 replicates in the 10K GAW15 data set without difficulty.

We have described the conceptual framework of CHORUS to highlight its strengths and its differences with other methods. We are exploring a range of enhancements including parallel computing that could enhance the efficiency and effectiveness of CHORUS further.

## Conclusion

Our results indicate that the information theoretic, KWII-based CHORUS approach has considerable promise as a method for GEI analysis of QT.

## Authors' contributions

PC carried out the development and implementation of the computational methods. LS carried out the statistical genetics analysis and interpretation. SL carried out the comparative genomics analysis. AZ was involved in the development of the computational analysis. MR conceived of the study and was involved all aspects of design and coordination. All authors read and approved the final manuscript.
